# Evaluation of Serum Cytokines Levels and the Role of Cannabidiol Treatment in Animal Model of Asthma

**DOI:** 10.1155/2015/538670

**Published:** 2015-05-25

**Authors:** Francieli Vuolo, Fabricia Petronilho, Beatriz Sonai, Cristiane Ritter, Jaime E. C. Hallak, Antonio Waldo Zuardi, José A. Crippa, Felipe Dal-Pizzol

**Affiliations:** ^1^Laboratório de Fisiopatologia Experimental, Programa de Pós-Graduação em Ciências da Saúde, Universidade do Extremo Sul Catarinense, 88806000 Criciúma, SC, Brazil; ^2^Programa de Pós-Graduação em Ciências da Saúde, Universidade do Sul de Santa Catarina, 88704900 Tubarão, SC, Brazil; ^3^Departamento de Neurociências e Ciências do Comportamento, Faculdade de Medicina de Ribeirão Preto, Universidade de São Paulo, 14049900 Ribeirão Preto, SP, Brazil

## Abstract

Asthma represents a public health problem and traditionally is classified as an atopic disease, where the allergen can induce clinical airway inflammation, bronchial hyperresponsiveness, and reversible obstruction of airways. Studies have demonstrated the presence of T-helper 2 lymphocytes in the lung of patients with asthma. These cells are involved in cytokine production that regulates immunoglobulin synthesis. Recognizing that T cell interaction with antigens/allergens is key to the development of inflammatory diseases, the aim of this study is to evaluate the anti-inflammatory potential of cannabidiol (CBD) in this setting. Asthma was induced in 8-week-old Wistar rats by ovalbumin (OVA). In the last 2 days of OVA challenge animals received CBD (5 mg/kg, i.p.) and were killed 24 hours after. The levels of IL-4, IL-5, IL-13, IL-6, IL-10, and TNF-*α* were determinate in the serum. CBD treatment was able to decrease the serum levels of all analyzed cytokines except for IL-10 levels. CBD seems to be a potential new drug to modulate inflammatory response in asthma.

## 1. Introduction

Atopic diseases, including dermatitis and asthma, sharing genetic and environmental risk factors, have their prevalence increased in recent decades and now affect approximately 20% of the population in developed countries [1, 2].

Asthma is a disorder of the conducting airways, which contract too much and too easily both spontaneously and in response to a wide range of exogenous and endogenous stimuli. This airway hyperresponsiveness is accompanied by enhanced sensory irritability of airways and increased mucus secretion. The different clinical expressions of asthma involve a variety of environmental factors that interact with the airways to cause acute and chronic inflammation. Several other factors contribute to this phenomenon, including smooth muscle contraction, edema, and remodeling of airways. Heterogeneity of asthma could also be related to different response to treatment [3].

Classically, asthma is characterized by chronic airway inflammation and remodeling, hyperresponsiveness, and increase of T-helper cell 2 levels- (Th2-) related cytokines [3]. To antagonize this response, several immunomodulatory strategies were tested in both preclinical and clinical settings. Cannabinoids are components of the* Cannabis sativa* (marijuana) plant and are known to exert potent anti-inflammatory, immunomodulatory, and analgesic effects through activation of cannabinoid-1 and cannabinoid-2 (CB1 and CB2) receptors located in the central nervous system (CNS) and immune cells, respectively [4]. Recently, a revision of Burstein has shown the involvement of CBD in control receptors involved in controlling inflammatory response, expression, and genetic transcription and reposted also functional effects in various diseases, colitis, sepsis-related encephalomyelitis, and inflammatory lung diseases [5]. It has also been demonstrated that CBD is well tolerated without significant effects even when chronically administered in humans [6]. The treatment of asthma remains problematic and often involves a combination of drugs, including corticosteroids, bronchodilators, leukotriene modifiers, and more recently target-directed therapies such as anti-IgE and anticytokines. All of such treatments have their strengths and limitations, such as the occurrences of side effects. Moreover, the patients with uncontrolled or partially controlled asthma, despite intensive treatment, remain a challenge. Thus, there is a clear need to explore new ways of managing and treating this respiratory disease [7].

The two most important cannabinoids, Δ9-tetrahydrocannabinol (Δ9-THC) and cannabidiol (CBD), were isolated and synthesized in the mid-1960s. CBD is the main nonpsychotropic cannabinoid and was shown to have beneficial effects in many pathological conditions, including neuropsychiatric disorders [8] and brain inflammatory diseases, without having significant activity on CB1 and CB2 receptors. Because of its beneficial effects in several pathologies and low toxicity in humans and other species [9], CBD represents a promising candidate for asthma treatment. In this regard, the present study was designed to evaluate the effects of CBD administration in inflammatory parameters (assessed by cytokines levels) in rats submitted to an asthma model.

## 2. Materials and Methods

### 2.1. Animals

Male adult Wistar rats (8 weeks old) were obtained from UNESC (Universidade do Extremo Sul Catarinense, Criciúma, Brazil) breeding colony. They were housed five per cage with food and water available ad libitum and were maintained on a 12 h light/dark cycle (lights on at 7:00 AM). All experimental procedures involving animals were performed in accordance with the NIH Guide for the Care and Use of Laboratory Animals, with the approval of Ethics Committee from Universidade do Extremo Sul Catarinense.

### 2.2. Study Design and Treatment

Rats were immunized by an i.p. injection of 10 *μ*g of chicken ovalbumin (OVA) in 100 *µ*L of aluminum hydroxide (alum) or alum alone. After 14 days, rats were boosted with OVA or alum. Seven days later, rats received aerosol challenges (30 min/for 3 d) with 1% OVA or saline.

CBD (THC Pharm, Frankfurt, Germany, and STI Pharm, UK) was suspended in 2% of polyoxyethylenesorbitan monooleate (Tween 80). The solutions were prepared immediately before use and were protected from light during the experimental session. CBD was administered i.p. once a day during the last two days of the OVA challenge at the dose of 5 mg/kg [10]. All treatments were administered in a volume of 1 mL/kg of CBD or vehicle. For this experiment, 21 rats were used, which were randomly divided into three groups: vehicle (control) (*n* = 7), OVA + vehicle (asthma control) (*n* = 7), and OVA + CBD (asthma + treatment) (*n* = 7). Blood samples were obtained 24 hours after the last challenge by decapitation to determine the levels of cytokines.

### 2.3. Serum Cytokine Assay

Serum interleukins IL-4, IL-5, IL-13, IL-6, and IL-10 and TNF-*α* were assayed with the Luminex xMap technology. Briefly, the specific antibodies for each cytokine were immobilised within microspheres through covalent bonds. After the analytic sample binds to the capture antibodies located on the surface of microspheres, the end detection is done via a third fluorescent marker, phycoerythrin connected to the detection antibody. The end result is a sandwich test through the microspheres. Cytokines were expressed as pg/mL serum.

### 2.4. Statistical Analysis

All data were reported as mean ± SEM. Statistical comparisons between experimental groups are using one-way ANOVA and post hoc Newman-Keuls tests. All statistical analyses were performed with the SPSS 17.0 (SPSS, Chicago, IL). Differences were considered significant when *p* < 0.05.

## 3. Results

**Figure 1:**
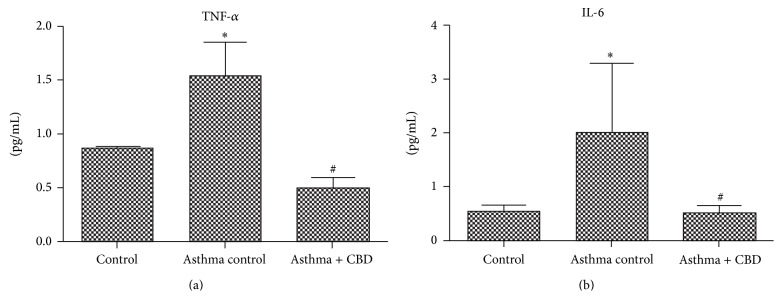
Effects of cannabidiol on Th1 profile cytokine levels. (a) TNF-*α* levels. (b) IL-6 levels. Bars represent means ± SD of 7 rats. ^*∗*^ *p* < 0.05 versus control vehicle, ^#^ *p* < 0.05 versus asthma control. ANOVA and post hoc Newman-Keuls tests.

**Figure 2:**
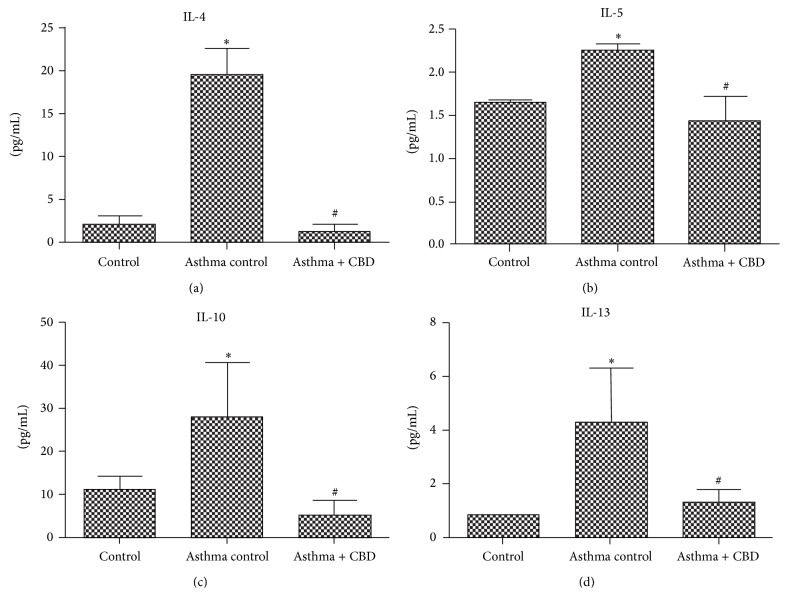
Effects of cannabidiol on Th2 profile cytokine levels. (a) IL-4 levels. (b) IL-5 levels. (c) IL-10 levels. (d) IL-13 levels. Bars represent means ± SD of 7 rats. ^*∗*^ *p* < 0.05 versus control vehicle, ^#^ *p* < 0.05 versus asthma control. ANOVA and post hoc Newman-Keuls tests.

We determined the levels of 6 cytokines implicated in asthma, which can be divided by the response profiles Th1 (TNF-*α* and IL-6) and Th2 (IL-4, IL-13, IL-10, and IL-5). Asthma induction resulted in an increase in all cytokines levels (Figures 1 and 2) when compared to control animals. CBD treatment was able to decrease both Th1 and Th2 cytokines (Figures 1 and 2).

## 4. Discussion

We here demonstrate a protective effect of CBD upon inflammatory response in an animal model of asthma; both Th1 and Th2 responses are blunted by CBD treatment.

This finding is consistent with a recent study in which CBD was shown to have potent immunosuppressive and anti-inflammatory properties. Therefore, we wonder whether CBD would be effective in controlling inflammation in a murine model of asthma as it has been reported in other models of inflammation [11]. The protective effects of CBD upon lung inflammation were demonstrated in several different models. Ribeiro and collaborators showed that treatment with CBD in a model of acute lung injury induced by lipopolysaccharide (LPS) decreased total lung resistance and elastance and improved several markers of inflammation [12]. However, inflammation associated with asthma is somewhat different from the observed in these models. Rats are sensitized by a large number of antigens that are not normally exposed to the environment, including OVA, which can elicit both Th1 and Th2 responses, which is different from LPS-induced inflammation [13].

CBD treatment significantly decreased the levels of cytokines involved in the immune response to an allergen, such as IL-4 and IL-5. IL-4 is responsible for the inhibition of Th1 cells differentiation and for Th2 cells differentiation and expansion and has an important role in IgE production. IL-4 was increased in patients with both atopic and nonatopic asthma [14, 15]. In addition, IL-4 seems to be a requisite for T lymphocytes to produce IL-5 [14–16]. IL-5 is essential for the maturation of eosinophils, and it accumulates in the lung during the inflammatory process of asthma [17].

Mucus hypersecretion is an important characteristic of asthma, which contributes to the exacerbation of symptoms. IL-13 is considered a major stimulus for this phenomenon [18]. We here demonstrate that IL-13 was reduced by CBD administration. IL-6 stimulates T cell proliferation, increasing IgE production dependent on IL-4. IL-6 levels are increased in sputum and systemic circulation in severe asthmatic patients, which can be responsible for the raise of C-reactive protein circulation in these patients [19]. CBD is also able to decrease IL-6 levels in an animal model. TNF-*α* is a major mediator of severe asthma, and it is demonstrated that soluble TNFR and antibodies against TNF-*α* [20, 21] are effective in animal models of asthma and CBD reduced the levels of TNF-*α* in our model.

In humans, several attempts to decrease inflammatory response in asthma have been tested. Anti-interleukin-4 and anti-interleukin-5 had been effective in reducing exacerbations and persistent eosinophilia in asthmatic patients [22]. In addition, anti-interleukin-9 and anti-interleukin-13 decreased symptoms associated with asthma [23, 24]. In this context, since CBD was used safely in humans, it is possible to suggest that a rapid transition to study its effects in humans is possible.

Until now, little was known about the effector cell of CBD in the immune system. Recently, Hedge and colleagues gave some important insights in this field. They demonstrated that CBD was able to induce myeloid-derived suppressor cells, and these cells suppressed T cell proliferation* ex vivo*. Additionally, this effect was dependent on the secretion of G-CSF by mast cells [25]. Furthermore, CBD is able to directly suppress T cell secretion of IL-2 and IFN-gamma [26]. These effects seem to be consistent with our findings of a protective effect on a murine model of asthma.

There are some limitations in the interpretation of the present data. Firstly, we do not have direct evidence of airways inflammation since we are not able to determine the effect of CBD in bronchoalveolar lavage fluid cytokines. Secondly, we do not measure lung function; thus we are not able to determine if CBD has any effect on flow obstruction in our model. Despite great relevance, we do believe that our results are of great importance. This is the first evidence of a beneficial effect of CBD in an animal model of asthma. In addition, we measure several different cytokines, and all the results are consistent with a significant anti-inflammatory effect of CBD.

In conclusion, we here demonstrate that the administration of CBD in an animal model of asthma could blunt the serum cytokine response to OVA in sensitized animals. These effects suggest a potential for a new asthma treatment since CBD controls the exaggerated inflammatory response observed in this model.
